# Evaluating the Quality of Cardiopulmonary Resuscitation in the Emergency Department by Real-Time Video Recording System

**DOI:** 10.1371/journal.pone.0139825

**Published:** 2015-10-02

**Authors:** Sheng Chen, Wenjie Li, Zhonglin Zhang, Hongye Min, Hong Li, Huiqi Wang, Yugang Zhuang, Yuanzhuo Chen, Chengjin Gao, Hu Peng

**Affiliations:** 1 Emergency Department, Shanghai Tenth People’s Hospital, Tongji University, School of Medicine, Shanghai, China; 2 Nursing Department, Shanghai Tenth People’s Hospital, Tongji University, School of Medicine, Shanghai, China; Azienda Ospedaliero-Universitaria Careggi, ITALY

## Abstract

**Objectives:**

To compare cardiopulmonary resuscitation (CPR) quality between manual CPR and miniaturized chest compressor (MCC) CPR. To improve CPR quality through evaluating the quality of our clinical work of resuscitation by real-time video recording system.

**Methods:**

The study was a retrospective observational study of adult patients who experienced CPR at the emergency department of Shanghai Tenth People’s Hospital from March 2013 to August 2014. All the performance of CPR were checked back by the record of “digital real-time video recording system”. Average chest compression rate, actual chest compression rate, the percentage of hands-off period, time lag from patient arrival to chest compression, time lag from patient arrival to manual ventilation, time lag from patient arrival to first IV establish were compared. Causes of chest compression hands-off time were also studied.

**Results:**

112 cases of resuscitation attempts were obtained. Average chest compression rate was over 100 compression per minute (cpm) in the majority of cases. However, indicators such as percentage of hands-off periods, time lag from patient arrival to the first manual ventilation and time lag from patient arrival to the first IV establish seemed to be worse in the manual CPR group compared to MCC CPR group. The saving of operators change time seemed to counteract the time spent on MCC equipment. Indicators such as percentage of hands-off periods, time lag between patient arrival to the first chest compression, time lag between patient arrival to the first manual ventilation and time lag from patient arrival to the first IV establish may influence the survival.

**Conclusion:**

Our CPR quality remained to be improved. MCC may have a potentially positive role in CPR.

## Introduction

The high incidence, low rate of survival, and unpredictability of cardiac arrest makes it a grave public health issue and a medical emergency. The application of cardiopulmonary resuscitation (CPR) plays a critical role in saving lives from cardiac arrest in and out of the hospital, and chest compression is the first part that plays a key role in CPR.

However, in spite of the formal and explicit specifications of chest compression presented in the resuscitation guidelines and examinations for the operators [1], various human and environmental factors in hospitals may result in unsatisfactory quality of chest compression and even varied outcomes [2–4].

Luckily, the defects or deficiency of operators can be figured out and corrected by using real-time video recording system and thus it may help to improve the survival [4]. In the meantime, the application of miniaturized chest compressor (MCC) may resolve the problems of physiological limits and the limited number of operators, providing continuous chest compression by minimizing no-chest compression intervals [5–7]. Significantly greater intrathoracic positive and negative pressures, diastolic intracranial pressure (ICP), cerebral perfusion pressure (CerPP), coronary perfusion pressure (CorPP), end-tidal PCO2 (ETCO_2_) and carotid blood flow have been also found in the domestic male pigs treated with MCC, with significantly lower compression depth and fewer rib fractures when compared with both the LUCAS and Thumper devices [8–9]. However, its actual effects remain to be under discussion [10–12].

The study was designed to improve our CPR quality by evaluating the quality of chest compressions and comparing the effects of manual-CPR and MCC-CPR by real-time video recording system.

## Methods

### 2.1 Study design

The study was conceived as a retrospective observational study. The inclusion criteria was: patients (> 18 years old) with cardiac arrest of all kinds of causes happening out of hospital or in hospital received cardiopulmonary resuscitation treatment at the emergency department (ED) of Shanghai Tenth People’ s Hospital from March 2013 to August 2014. The exclusion criteria was: the video records were incomplete or failed to obtain data required; Patients were diagnosed as “clinical death” before or at the time of hospital arrival; Family refused to participate in the study. The family was consulted and provided informed consent on arrival because of patients compromised capacity to consent. Video recording would be deleted completely without agreement. The ethics committee of Shanghai Tenth People’s Hospital approved the observational study and the consent procedure.

### 2.2 Data collection

Three real-time video recording systems (DS-8000 video network hard disk recorder, Hikvision Company, Hangzhou, China) installed in the CPR room of ED recorded the individual steps and performance of CPR in all the cases arriving at the CPR room from different directions. The events were automatically time stamped and saved in the hard disk. Each patient’s data was also extracted and was stored in the medical documents if the integrity of the video was satisfied.

The following data were collected from the special medical documents for each patient: age, sex, history diseases, the time and causes of cardiac arrest, initial rhythm and survival to be hospitalized.

All video records were reviewed for CPR quality by focusing on the following domains: manual CPR or MCC (MCC 100, Sunlife Science, Hongkong) CPR as main chest compression technique, average chest compression rate (AVCR), actual chest compression rate (ACCR), time lag from patient arrival to first chest compression, time lag from patient arrival to first manual ventilation, time lag from patient arrival to first IV establish, percentage of hands-off time in total chest compression time, causes of chest compression hands-off time, survival to be hospitalized. MCC group included patients applied with MCC after arriving at the hospital; the chest compression out-of-hospital was not counted. AVCR = (N: total chest compression counts) / (T1: total chest compression time). ACCR = N / (T1—T2) (T2: hands-off time). Hands-off time was defined as the time that was longer than 1s between two consecutive compressions. Percentage of hands-off time = T2 / T1.

### 2.3 Statistical Analysis

All data was analyzed by using SPSS19.0 (SPSS Inc. Chicago, IL, USA) software and for subsequent analysis of data. Counts (percentage) and 95% CI (binomial CIs) were used for count data. Mean and 95% Confidence (95% CI) intervals were used for normally distributed data, and median and interquartile ranges were used for non-parametric continuous data.

## Results

### 3.1 Demographic information

From March 2013 to August 2014, 112 cases of resuscitation attempts were obtained, 3 cases of resuscitation video excluded due to recording device dysfunction lost. The demographic information and basic data were shown in **Table 1**. The patients were divided into manual CPR group and MCC CPR group.

**Table 1:** Demographic and Descriptive Clinical Data.

	Manual CPR Group (n = 50)	MCC CPR Group (n = 62)	Total (n = 112)
**Age (years)**	61 (56.61–64.51)	59 (54.96–62.10)	59 (56.82–62.05)
**Sex (male / female)**	30 (60.0) (45.18–73.59) /20 (40.0) (26.41–54.82)	38 (61.3) (48.06–73.40) / 24 (38.7) (26.60–51.94)	68 (60.7) (51.04–69.81) / 44 (39.3) (30.19–48.96)
**History diseases**			
Coronary heart disease	25 (50.0) (35.53–64.47)	28 (45.2) (32.48–58.32)	53 (47.3) (37.81–56.98)
High blood pressure	25(50.0) (35.53–64.47)	35 (56.5) (43.26–69.01)	60 (53.6) (43.90–63.05)
Diabetes mellitus	16 (32.0) (19.52–46.70)	21 (33.9) (22.33–47.01)	37 (33.0) (24.44–42.56)
Others/unknown	11 (22.0) (11.53–35.96)	13 (21.0) (11.66–33.18)	24 (21.4) (14.24–30.19)
**Time of cardiac arrest**			
Morning (06:00AM-12:00PM)	14 (28.0) (16.23–42.49)	18 (29.0) (18.20–41.95)	32 (28.6) (20.43–37.88)
Afternoon (12:00PM-06:00PM)	19 (38.0) (24.65–52.82)	23 (37.1) (25.16–50.31)	42 (37.5) (28.53–47.15)
Evening (06:00PM-12:00AM)	10 (20.0) (10.03–33.72)	8 (12.9) (5.74–23.85)	18 (16.1) (9.81–24.21)
Night (12:00AM-06:00AM)	7 (14.0) (5.82–26.74)	13 (21.0) (11.66–33.18)	20 (17.9) (11.26–26.22)
**Cause of cardiac arrest**			
Toxin	4 (8.0) (2.22–19.23)	7 (11.3) (4.66–21.89)	11 (9.8) (5.01–16.89)
Trauma	7 (14.0) (5.82–26.74)	14 (22.6) (12.93–34.97)	21 (18.8) (12.00–27.22)
Asphyxia	5 (10.0) (3.33–21.81)	9 (14.5) (6.86–25.78)	14 (12.5) (7.01–20.08)
Cerebral vascular disease	30 (60.0) (45.18–73.59)	25 (40.3) (28.05–53.55)	55 (49.1) (39.54–58.73)
others/unknown	4 (8.0) (2.22–19.23)	7 (11.3) (4.66–21.89)	11 (9.8) (5.01–16.89)
**Initial rhythm**			
Asystole /pulseless electric activity	39 (78.0) (64.04–88.47)	46 (74.2) (61.50–84.47)	85 (75.9) (66.90–83.47)
Ventricular fibrillation	9 (18.0) (8.58–31.44)	12 (19.3) (10.42–31.37)	21 (18.8) (12.00–27.22)
others/ unknown	2 (4.0) (0.49–13.71)	4 (6.4) (1.79–15.7)	6 (5.3) (1.99–11.30)

Note: The patients were divided into manual CPR and MCC CPR group depending on whether the patients were applied with MCC after arriving at the hospital, the chest compression out-of-hospital was not counted. Data are presented as counts (percentage) (95% CI) of population except age [mean (95% CI)] (parametric).

### 3.2 CPR parameters in different chest compression approach

The CPR parameters used during the cardiac arrest of all patient were shown in **Table 2**. Average chest compression rate was over 100 compression per minute (cpm) in the majority of cases after calibration of hands-off time. However, indicators such as percentage of hands-off periods, time lag from patient arrival to the first manual ventilation and time lag from patient arrival to the first IV establish seemed to be worse in the manual CPR group compared to MCC CPR group.

**Table 2:** CPR Parameters During Cardiac Arrest Episodes.

	Manual CPR Group (n = 50)	MCC CPR Group (n = 62)	Total (n = 112)
**AVCR (cpm)**	85.5 (70.75–95.50)	86.0 (70.00–97.00)	86.0 (70.25–96.25)
**ACCR (cpm)**	104.0 (100.00–108.00)	100.0 (100.00–100.00)	100.0 (100.00–103.00)
**Percentage of hands-off periods (%)**	10.0 (8.00–12.00)	9.0 (6.75–11.00)	9.0 (8.00–11.00)
**Time lag from patient arrival to the first chest compression (s)**	18.0 (14.75–20.00)	19.0 (14.75–21.00)	18.0 (15.00–20.75)
**Time lag from patient arrival to the first manual ventilation (s)**	58.0 (45.75–67.50)	55.5 (43.00–69.00)	56.5 (44.00–69.00)
**Time lag from patient arrival to the first IV establish (s)**	190.5 (172.75–219.25)	188.5 (170.5–210.25)	190.0 (171.00–212.00)
**Survival to be hospitalized (n)**	10 (20.0) (10.03–33.72)	19 (30.6) (19.56–43.65)	29 (25.9) (18.08–35.03)

Note: The patients were divided into manual CPR group and MCC CPR group depending on whether the patients were applied with MCC after arriving at the hospital, the chest compression out-of-hospital was not counted. cpm: compression per minute. AVCR = (N: total chest compression counts) / (T1: total chest compression time). ACCR = N / (T1—T2) (T2: hands-off time). Hands-off time was defined as the time that was longer than 1s between two consecutive compressions. Percentage of hands-off time = T2 / T1. Data was presented as median (interquartile ranges) (non-parametric) except survival [counts (percentage) (95% CI)].

### 3.3 Various reasons caused hands-off time

The causes and length of hands-off time during chest compression in our study were analyzed (**Table 3**). In the MCC CPR group, MCC equipment did cost more hands-off time. Interestingly, the saving of operators change time seemed to counteract the time spent on MCC equipment.

**Table 3:** Causes and length of hands-off time.

	Percentage of hands-off periods (%)			Survival (n)		
	Manual CPR Group	MCC CPR Group	Total	Manual CPR Group	MCC CPR Group	Total
**Defibrillation**						
**Yes (n = 25)**	10 (7.25–11.00)	8 (6.00–10.00)	9 (7.00–11.00)	3 (12.0) (2.55–31.22)	3 (12.0) (2.55–31.22)	6 (24.0) (9.36–45.13)
**No (n = 87)**	10.5 (8.00–12.00)	9 (6.50–11.00)	10 (8.00–12.00)	7 (8.0) (3.30–15.88)	16 (18.39) (10.89–28.14)	23 (26.4) (17.55–36.98)
**Intubation**						
**Yes (n = 57)**	12 (8.00–13.00)	9 (8.00–11.00)	10 (8.00–12.00)	3 (5.3) (1.10–14.62)	10 (17.5) (8.75–29.91)	13 (22.8) (12.74–35.84)
**No (n = 55)**	10 (8.00–11.00)	8 (5.00–10.00)	9 (7.00–11.00)	7 (12.7) (5.27–24.48)	9 (16.4) (7.77–28.80)	16 (29.1) (17.63–42.90)
**Operators Change**						
**Yes (n = 29)**	11 (8.50–12.00)	N/A	11 (8.50–12.00)	7 (24.1) (10.30–43.54)	N/A	7 (24.1) (10.30–43.54)
**No (n = 83)**	10 (8.00–12.00)	9 (6.75–11.00)	9 (7.00–11.00)	3 (3.6) (0.75–10.20)	19 (22.9) (14.38–33.42)	22 (26.5) (17.42–37.34)

Note: Hands-off time was defined as the time that was longer than 1s between two consecutive compressions. Percentage of hands-off time = T2 (T2: hands-off time) / T1 (T1: total chest compression time). Percentage of hands-off time was presented as median (interquartile ranges) (non-parametric) and survival was presented as counts (percentage) (95% CI).

### 3.4 CPR parameters during cardiac arrest episodes

The CPR parameters were also compared between survival group and non-survival group (**Table 4**). Indicators such as percentage of hands-off periods, time lag between patient arrival to the first chest compression, time lag between patient arrival to the first manual ventilation and time lag from patient arrival to the first IV establish may influence the survival.

**Table 4:** CPR Parameters During Cardiac Arrest Episodes.

	Survival Group (n = 29)	Non-survival Group (n = 83)
**AVCR (cpm)**	85.0 (72.00–96.00)	86.0 (70.00–97.00)
**ACCR (cpm)**	100.0 (100.00–102.00)	100.0 (100.00–104.00)
**Percentage of hands-off periods (%)**	8.0 (5.00–8.50)	10.0 (9.00–12.00)
**Time lag between patient arrival to the first chest compression (s)**	16.0 (14.00–19.50)	18.0 (15.00–21.00)
**Time lag between patient arrival to the first manual ventilation (s)**	49.0 (38.50–69.00)	57.0 (46.00–69.00)
**Time lag from patient arrival to the first IV establish (s)**	185.0 (164.50–195.50)	197.0 (175.00–220.00)

Note: The patients were divided into survival and non-survival group depending on the survival to be hospitalized. cpm: compression per minute. AVCR = (N: total chest compression counts) / (T1: total chest compression time). ACCR = N / (T1—T2) (T2: hands-off time). Hands-off time was defined as the time that was longer than 1s between two consecutive compressions. Percentage of hands-off time = T2 / T1. Data was presented as median (interquartile ranges) (non-parametric).

## Discussion

In this study, observational retrospective study a real-time video recording system was used to learn the overall characteristics of patients applied with manual or mechanical CPR, the quality and relevant factors of CPR performance in our department, trying to reflect the status of CPR in hospitals of better level in China objectively, and to improve the quality of CPR.

We found that asystole / pulseless electric activity appeared to be the most frequent initial rhythm, instead of ventricular fibrillation (VF), the initial rhythm in the majority of out-of-hospital cardiac arrests [13]. The fact was also noted in other recent studies, in which 12.7%-25% of initial in-hospitalal rhythm was VF while the percentage of VF as out-of-hospital initial rhythm could be 40% [14–17]. On one hand, it may result from the implemented defibrillation out of the hospital in those patients with VF. On the contrary, VF diminishes rapidly over time, and there may exist a delay in the transportation to the hospital. Also, most of the patients staying in the emergency department were presented with multiple organ dysfunction syndromes (MODS) and had a low probability of VF when the cardiac arrest happened compared to those out of the hospital.

From previous studies it is obvious that good-quality CPR improves the chances of survival and quality of life for cardiac arrest patients [18]. The most significant changes in CPR guidelines 2010 were made to simplify CPR instruction and increase the number of chest compressions delivered per minute and reduce interruptions in chest compressions during CPR [19]. However, the quality of CPR was still worrying [20–23]. Failure in chest compression promptly and continuously could make recovery of heart rate and oxygen supply to the brain and other vital tissue more difficult. One of the most apparent problems was the social environment, for it is common in most hospitals in the region where there is overcrowding in the CPR room. The establishment of closed or semi-open CPR rooms and rationalization of emergency physician’s assignment and management may improve the situation.

However, at the same time, we should recognize some limitations of human beings. Excessive and unreasonable consumption of human resources may result in shortage. Fatigue and weakened effects could be obtained after the first minute without realization of operators [24–25], and it increases the difficulty in transportation and treatment of patients. Moreover, it was reported that little blood flow as much as 10%~20% normal volume could be produced to supply for heart and 20%~30% for brain through traditional manual chest compressions [26]. Thus the advanced technologies should be applied.

The application of Weil MCC could ① achieve the same perfusion with half the compression depth and strictly maintain the compression/relax ratio at 1:1; ② get better performance in nervous system after recovery; ③ result in decreased occurrence of complications such as rib fractures in a Thumper-controlled pig experiment [5]; ④ make it possible to apply compression and defibrillation simultaneously. However, it should be pointed out that defibrillation efficacy is maximal when electrical shock is delivered in the upstroke phase of mechanical chest compression. Otherwise, defibrillation success rate could be even lower [27].

In this observational study, we can easily make sense from the results that application of MCC could reduce the percentage of hands-off periods in resuscitation time, and medical resources as well. Accordingly, time for operators change could be saved, time lag from patient arrival to the first manual ventilation and time lag from patient arrival to the first IV establish could be cut down, which could be elements that influence CPR quality, even patients survival.

Interestingly, even if the operators didn’t change in the manual CPR group, the survival to be hospitalized still appeared to be improved in the MCC CPR group. The results may be associated with depth of compression, timely intubation and defibrillation.

As a department in a hospital listed in the Supreme hospitals in the developed area in China, it has sufficient equipment and is thought as a representative for the objective condition of emergency departments in high-level hospitals. In the study, we have observed the actual characteristics of major CPR features as well as deficiencies in the management, transportation and CPR operation in our department. Luckily, we considered most of them could be improved through a variety of means. We explored the potentially positive role of the MCC applied in CPR in the study and tried to establish the basis for further extensive research.

There were several limitations to our study. A primary limitation was that the contact information of the patients was incomplete so that we failed to collect the data of cerebral function and outcomes after discharge from the hospital. Second, we didn’t evaluate the precise depth, placement of chest compression and the lag from cardiac arrest occurrence to arrival, for they could not be obtained from the video recording system, and the lag from cardiac arrest occurrence to arrival as well. The third limitation of our study was that it was an observational study, and that cause and effect cannot be established. The fourth limitation is that the study was single-center. Multi-center randomized controlled trials (RCT) with larger sample size and prospective studies of the effects of suggested measures could further enhance our knowledge in this aspect.

## References

[1] 2010 American Heart Association Guidelines for Cardiopulmonary Resuscitation and Emergency Cardiovascular Care Circulation, Volume 112, Issue 24 Supplement; 12 13, 2010.

[2] Sutton R M, Niles D, French B, Maltese M R, Leffelman J, EilevstjØnn J, et al First quantitative analysis of cardiopulmonary resuscitation quality during in-hospital cardiac arrests of young children. Resuscitation 2014; 85(1): 70–74. 10.1016/j.resuscitation.2013.08.014 23994802 PMC3877702

[3] Yang Z, Li H, Yu T, Chen C, Xu J, Chu Y, et al Quality of chest compressions during compression-only CPR: a comparative analysis following the 2005 and 2010 American Heart Association guidelines. The American journal of emergency medicine 2014; 32(1): 50–54. 10.1016/j.ajem.2013.09.043 24210889

[4] Jiang C, Zhao Y, Chen Z, Chen S, Yang X. Improving cardiopulmonary resuscitation in the emergency department by real-time video recording and regular feedback learning. Resuscitation 2010; 81(12): 1664–1669. 10.1016/j.resuscitation.2010.06.023 20727657

[5] Ristagno G, Castillo C, Tang W, Sun S, Bisera J, Weil M H. Miniaturized mechanical chest compressor: A new option for cardiopulmonary resuscitation. Resuscitation 2008; 76(2): 191–197. 17728044 10.1016/j.resuscitation.2007.07.004

[6] Wang H C, Chiang W C, Chen S Y, S Y, Ke Y L, et al Video-recording and time-motion analyses of manual versus mechanical cardiopulmonary resuscitation during ambulance transport. Resuscitation 2007; 74(3): 453–460. 17386966 10.1016/j.resuscitation.2007.01.018

[7] Bonnemeier H, Simonis G, Olivecrona G, Weidtmann B, Götberg M, Weitz G, et al Continuous mechanical chest compression during in-hospital cardiopulmonary resuscitation of patients with pulseless electrical activity. Resuscitation 2011; 82(2): 155–159. 10.1016/j.resuscitation.2010.10.019 21126816

[8] Chen W, Weng Y, Wu X, Sun S, Bisera J, Weil M H, Tang W. The effects of a newly developed miniaturized mechanical chest compressor on outcomes of cardiopulmonary resuscitation in a porcine model. Critical care medicine 2012; 40(11): 3007–3012. 10.1097/CCM.0b013e31825d924d 23080437

[9] Xu J, Hu X, Yang Z, Wu X, Bisera J, Sun S, Tang W. Miniaturized mechanical chest compressor improves calculated cerebral perfusion pressure without compromising intracranial pressure during cardiopulmonary resuscitation in a porcine model of cardiac arrest. Resuscitation 2014; 85(5): 683–688. 10.1016/j.resuscitation.2014.01.014 24463224

[10] Westfall M, Krantz S, Mullin C, Kaufman C. Mechanical Versus Manual Chest Compressions in Out-of-Hospital Cardiac Arrest: A Meta-Analysis*. Critical care medicine 2013; 41(7): 1782–1789. 10.1097/CCM.0b013e31828a24e3 23660728

[11] Brooks S C, Bigham B L, Morrison L J. Mechanical versus manual chest compressions for cardiac arrest The Cochrane Library 2011. 10.1002/14651858.CD007260.pub2 21249689

[12] Ong M E, Mackey K E, Zhang Z C, Tanaka H, Ma M H M, Swor R, Shin S D. Mechanical CPR devices compared to manual CPR during out-of-hospital cardiac arrest and ambulance transport: a systematic review. Scand J Trauma Resusc Emerg Med 2012; 20(1): 39. 22709917 10.1186/1757-7241-20-39 PMC3416709

[13] Zevitz M E. Ventricular Fibrillation. E-Medicine CME 2003.

[14] Kaye W, Mancini ME. Retention of cardiopulmonary resuscitation skills by physicians, registered nurses, and the general public. Crit Care Med 1986; 14: 620–2. 3720312 10.1097/00003246-198607000-00007

[15] Broomfield R. A quasi-experimental research to investigate the retention of basic cardiopulmonary resuscitation skills and knowledge by qualified nurses following a course in professional development. J Adv Nursing 1996; 23: 1016–23. 10.1111/j.1365-2648.1996.tb00084.x 8732531

[16] Peberdy MA, Kaye W, Ornato JP, Larkin GL, Nadkarni V, Mancini ME, Berg RA, Nichol G, Lane-Trultt T. Cardiopulmonary resuscitation of adults in the hospital: a report of 14720 cardiac arrests from the National Registry of Cardiopulmonary Resuscitation. Resuscitation. 2003; 58: 297–308. 12969608 10.1016/s0300-9572(03)00215-6

[17] Abella BS, Alvarado JP, Myklebust H, Edelson D P, Barry A, O’Hearn N, et al Quality of Cardiopulmonary Resuscitation During In-Hospital Cardiac Arrest. JAMA 2005; 293(3): 305–310. 15657323 10.1001/jama.293.3.305

[18] Cunningham L M, Mattu A, O'Connor R E, Brady W J. Cardiopulmonary resuscitation for cardiac arrest: the importance of uninterrupted chest compressions in cardiac arrest resuscitation. The American journal of emergency medicine 2012; 30(8): 1630–1638. 10.1016/j.ajem.2012.02.015 22633716

[19] Berg R A, Hemphill R, Abella B S, Aufderheide T P, Cave D M. Hazinski M F, et al Part 5: Adult basic life support 2010 American Heart Association guidelines for cardiopulmonary resuscitation and emergency cardiovascular care. Circulation 2010; 122 (18 suppl 3): S685–S705. 10.1161/CIRCULATIONAHA.110.970939 20956221

[20] Sutton R M, Niles D, French B, Maltese M R, Leffelman J, EilevstjØnn J, et al First quantitative analysis of cardiopulmonary resuscitation quality during in-hospital cardiac arrests of young children. Resuscitation 2014; 85(1): 70–74. 10.1016/j.resuscitation.2013.08.014 23994802 PMC3877702

[21] Ødegaard S, Olasveengen T, Steen P A, Kramer-Johansen J. The effect of transport on quality of cardiopulmonary resuscitation in out-of-hospital cardiac arrest. Resuscitation 2009; 80(8): 843–848. 10.1016/j.resuscitation.2009.03.032 19477573

[22] Sutton R M, Wolfe H, Nishisaki A, Leffelman J, Niles D, Meaney P.A., et al Pushing harder, pushing faster, minimizing interruptions… but falling short of 2010 cardiopulmonary resuscitation targets during in-hospital pediatric and adolescent resuscitation. Resuscitation 2013; 84(12): 1680–1684. 10.1016/j.resuscitation.2013.07.029 23954664 PMC3825766

[23] Meaney P A, Bobrow B J, Mancini M E, Christenson J, de Caen A R, Bhanji F, et al Cardiopulmonary resuscitation quality: improving cardiac resuscitation outcomes both inside and outside the hospital a consensus statement from the American Heart Association. Circulation 2013; 128(4): 417–435. 10.1161/CIR.0b013e31829d8654 23801105

[24] Tipton M, David G, Eglin C, Golden F. Basic life support on small boats at sea. Resuscitation 2007; 75(2): 332–337. 17574722 10.1016/j.resuscitation.2007.04.027

[25] Aufderheide T P, Pirrallo R G, Yannopoulos D, Klein J P, von Briesen C, Sparks C W, et al Incomplete chest wall decompression: a clinical evaluation of CPR performance by EMS personnel and assessment of alternative manual chest compression–decompression techniques. Resuscitation 2005; 64(3): 353–362. 15733766 10.1016/j.resuscitation.2004.10.007

[26] Kern K B. Coronary perfusion pressure during cardiopulmonary resuscitation. Baillieres Clin Anaesth 2000; 14: 59–609.

[27] Li Y, Wang H, Cho J H, Quan W, Freeman G, Bisera J, et al Defibrillation delivered during the upstroke phase of manual chest compression improves shock success. Critical care medicine 2010; 38(3): 910–915. 10.1097/CCM.0b013e3181cc4944 20042857

